# Implications of blood type in personalised microbiome therapy

**DOI:** 10.1002/ctm2.1618

**Published:** 2024-03-11

**Authors:** Yue Zhang, Ranko Gacesa, Jingyuan Fu

**Affiliations:** ^1^ Department of Genetics University of Groningen University Medical Center Groningen Groningen The Netherlands; ^2^ Department of Gastroenterology and Hepatology University Medical Center Groningen Groningen The Netherlands; ^3^ Department of Pediatrics University of Groningen, University Medical Center Groningen Groningen The Netherlands

## 1 INTRODUCTION

The critical role the gut microbiome can play in personalised medicine is deeply rooted in the symbiotic relationship between the host and gut microorganisms, a relationship shaped by their coevolution and extensive host–microbe metabolic and immune interactions. It is hypothesised that host genetics can determine the chemical and physical environment that gut microorganisms inhabit, including nutrient availability and immune system thresholds, but genetics‐based evidence and mechanistic understanding are still lacking. We recently carried out a genome‐wide association study between human genetics and gut microbial structural variations (SVs),^1^ which shed light on how blood type A sugar fuels gut microbes and promotes human health. Here, we summaries our key findings and elaborate on the implications for clinical practice.

## 2 CAUSAL GENE DISCOVERY VIA THE LENS OF STRUCTURAL VARIATION

Heritability of the gut microbiome has been observed in animals and humans. In recent years, genome‐wide association studies on microbial species abundance level have yielded several robust associations with host genetic loci. However, the genetic diversity within individual species can complicate mechanistic interpretation.^2^ Specifically, bacterial SVs, segments of the bacterial genome of different lengths, are an essential microbial adaptive mechanism. SVs have been found to play a crucial role in enhancing microbial survival, carbohydrate utilisation, metabolism and even pathogenic potential.^3^ We were therefore motivated to identify microbial genes under host control via the lens of microbial SVs.

## 3 A MICROBIAL CARBON SOURCE DERIVED FROM BLOOD TYPE‐LINKED CARBOHYDRATES

**FIGURE 1:**
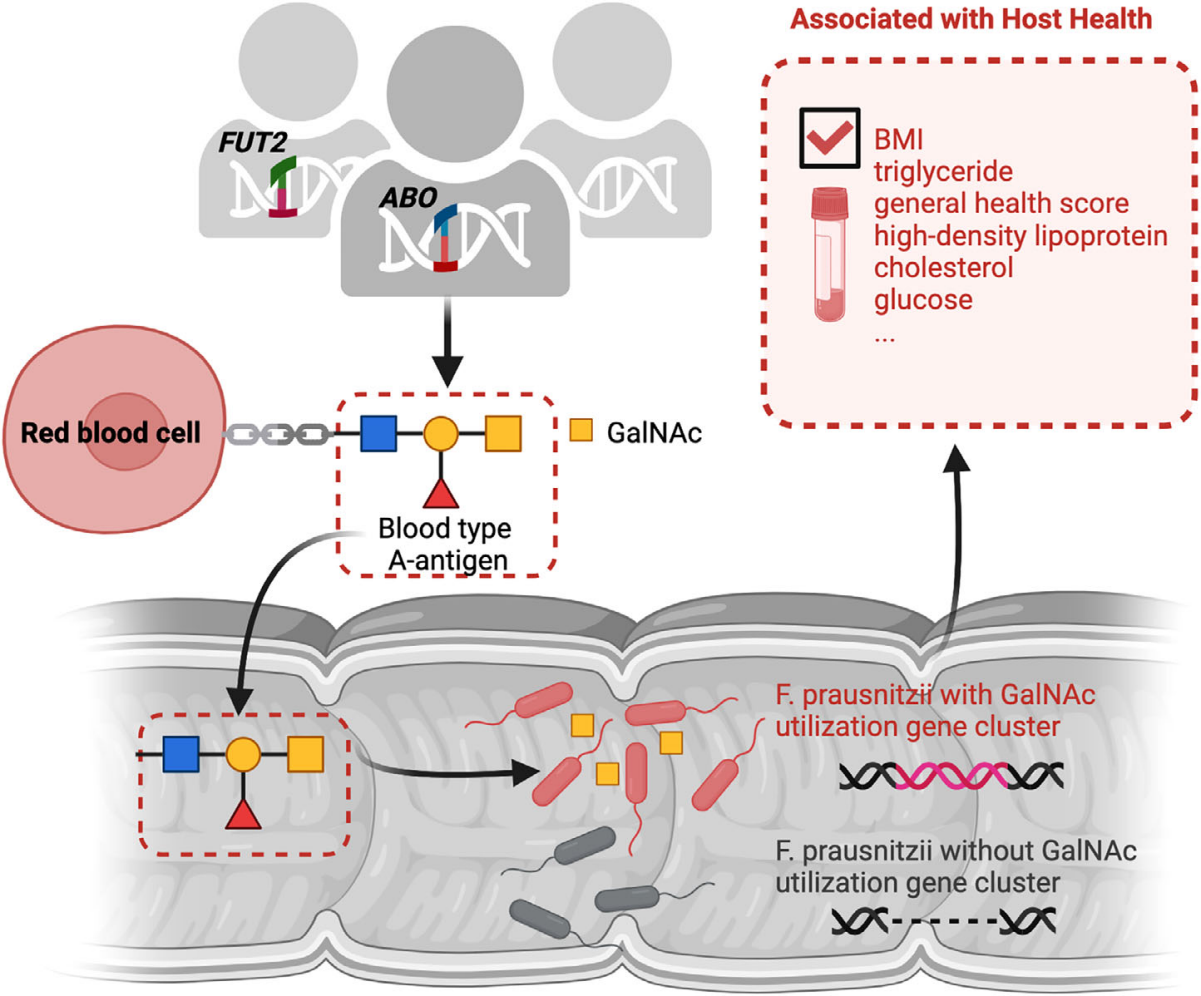
GalNAc utilisation by the gut microbiome. Zhernakova et al. studied associations between genetic variants in the human genome and those in the gut metagenome, providing functional insights into GalNAc utilisation by gut microbes. The abundance of GalNAc in intestinal mucus is determined by an individual's *ABO* and *FUT2* genotypes, and *Faecalibacterium prausnitzii* with the GalNAc pathway show positive association with the host's cardiometabolic health.

Our study identified striking association between the human *ABO* locus and a specific SV region in the gut microbe *Faecalibacterium prausnitzii*. The *ABO* gene encodes a transferase that determines the terminal sugars of blood type antigens: GalNAc for blood type A and galactose for blood type B. In people who are also *FUT2* secretors, these blood type antigens can be freed and released into body fluid, including into intestinal mucus. Interestingly, the SV region of *F. prausnitzii* that we identified contains a GalNAc utilisation gene cluster, and we further found that the presence rate of this cluster was significantly higher in blood type A/AB individuals with *FUT2* secretor status. Our study also revealed mechanistic evidence that gut microbes can utilise blood type‐linked sugar as nutrients for growth, and we also found evidence for a beneficial impact on microbial niche and host cardiometabolic health (Figure 1). The study was the first human study to move beyond established associations between host genetics and gut microbial abundance to pinpointing potential causal genes of both human genome and the gut microbiome, bridging the gap from species abundance to functionality.

## 4 IMPLICATION IN CLINICAL TRANSLATION

The identified association involves the *ABO* gene and *F. prausnitzii*, both of which have been implicated in human health. The *ABO* genotype has been associated with many diseases and traits, including venous thromboembolism, lipid levels, cardiometabolic phenotypes and susceptibility to infectious diseases such as dengue, malaria and even COVID‐19.^4^, ^5^ Its pleiotropic effect has also been observed at molecular levels, such as in cardiovascular‐disease‐related proteins.^6^ *F. prausnitzii* is a major fibre fermenter in our intestine that produces a health‐promoting short‐chain fatty acid called as butyrate. The beneficial role of *F. prausnitzii* has been reported in all kinds of human diseases, ranging from cardiometabolic, immune and mental diseases to cancers. Pre‐clinical experiments have shown it has a beneficial role in shaping the intestinal microbial flora, restoring metabolic homeostasis and alleviating inflammation.^7^ However, *F. prausnitzii* is a strictly anaerobic microbe, and development of *F. prausnitzii* as a next‐generation probiotic still faces some technical hurdles.^8^ Approaches to manipulate its intestinal abundance still largely rely on dietary intervention (e.g., high fibre intake) or faecal microbiota transplantation (FMT). FMT has now been approved as an effective approach in treating *Clostridium difficile* infections,^9^ and its application is emerging for the treatment of other diseases and in combination with other treatment to enhance therapeutic outcome. The most notable example thus far is that FMT can enhance efficacy of immunotherapy,^10^, ^11^ and *F. prausnitzii* has been shown to abrogate intestinal toxicity and promote immunity.^12^ Thus, colonisation with the right *F. prausnitzii* strain for the patient is crucial. Blood compatibility is critical in solid organ transplantation; our results suggest that it might also be necessary to match blood type to a specific strain of *F. prausnitzii* to promote engraftment and colonisation of *F. prausnitzii*. In other ways, our study provides evidence of the need for personalised microbiome‐based medicine.

## 5 LIMITATIONS AND FUTURE PERSPECTIVES

This study provides mechanistic insights into microbial utilisation of carbohydrates produced by the human genome, demonstrating a positive health impact in individuals with A blood type and *FUT2* secretor status. However, it remains unclear whether their improved health is due to the direct effect of GalNAc utilisation or a secondary effect of the enhanced growth of the beneficial bacterium *F. prausnitzii*. Future research needs to construct comprehensive in vivo models that can unravel the causal relationship, particularly the interaction between A‐antigen, *F. prausnitzii*, and host health outcomes.

In addition to the *ABO* locus, our study reported >200 suggestive associations with small to modest effect size. Their underlying biological mechanisms and potential physiological value await detailed understanding. Future research thus calls for cross‐disciplinary approaches such as the integration of omics technologies and statistical tools in combination with in vivo and in vitro approaches.

## AUTHOR CONTRIBUTIONS

J.F. supervised the whole writing process and revised the manuscript. Y.Z. wrote the manuscript with critical reading and feedback from R.G.

## ETHICS STATEMENT

All authors have been personally and actively involved in substantial work leading to the paper and will take public responsibility for its content.
